# SP-CHAP, an endolysin with enhanced activity against biofilm pneumococci and nasopharyngeal colonization

**DOI:** 10.1128/mbio.00069-24

**Published:** 2024-03-12

**Authors:** Adit B. Alreja, Amanda E. Appel, Jinyi C. Zhu, Sean P. Riley, Norberto Gonzalez-Juarbe, Daniel C. Nelson

**Affiliations:** 1 Institute for Bioscience and Biotechnology Research, University of Maryland, Rockville, Maryland, USA; 2 Department of Infectious Diseases and Genomic Medicine, J. Craig Venter Institute, Rockville, Maryland, USA; 3 Department of Veterinary Medicine, University of Maryland, College Park, Maryland, USA; 4 Department of Cell Biology and Molecular Genetics, University of Maryland, College Park, Maryland, USA; Mississippi State University, Mississippi State, Mississippi, USA

**Keywords:** endolysin, CHAP domain, bacteriophage, pneumococcus, antibiotic resistance

## Abstract

*Streptococcus pneumoniae* (*Spn*), a Gram-positive bacterium, is responsible for causing a wide variety of invasive infections. The emergence of multi-drug antibiotic resistance has prompted the search for antimicrobial alternatives. Phage-derived peptidoglycan hydrolases, known as endolysins, are an attractive alternative. In this study, an endolysin active against *Spn*, designated SP-CHAP, was cloned, produced, purified, biochemically characterized, and evaluated for its antimicrobial properties. Cysteine, histidine-dependent amidohydrolase/peptidase (CHAP) domains are widely represented in bacteriophage endolysins but have never previously been reported for pneumococcal endolysins. Here, we characterize the first pneumococcal endolysin with a CHAP catalytic domain. SP-CHAP was antimicrobial against all *Spn* serovars tested, including capsular and capsule-free pneumococci, and it was found to be more active than the most widely studied pneumococcal endolysin, Cpl-1, while not affecting various oral or nasal commensal organisms tested. SP-CHAP was also effective in eradicating *Spn* biofilms at concentrations as low as 1.56 µg/mL. In addition, a *Spn* mouse nasopharyngeal colonization model was employed, which showed that SP-CHAP caused a significant reduction in *Spn* colony-forming units, even more than Cpl-1. These results indicate that SP-CHAP may represent a promising alternative to combating *Spn* infections.

**IMPORTANCE:**

Considering the high rates of pneumococcal resistance reported for several antibiotics, alternatives are urgently needed. In the present study, we report a *Streptococcus pneumoniae*-targeting endolysin with even greater activity than Cpl-1, the most characterized pneumococcal endolysin to date. We have employed a combination of biochemical and microbiological assays to assess the stability and lytic potential of SP-CHAP and demonstrate its efficacy on pneumococcal biofilms *in vitro* and in an *in vivo* mouse model of colonization. Our findings highlight the therapeutic potential of SP-CHAP as an antibiotic alternative to treat *Streptococcus pneumoniae* infections.

## OBSERVATION

Endolysins offer an alternative to conventional antibiotics in addressing the emergence of multi-drug-resistant bacteria. These enzymes, encoded by bacteriophages, induce cell lysis upon synthesis at the end of the replication cycle and are not susceptible to efflux pumps, penicillin-binding proteins, or other common mechanisms of antimicrobial resistance (1, 2). Moreover, the development of any resistance to endolysins has not been reported (3). *Streptococcus pneumoniae* (*Spn*), a Gram-positive nasopharyngeal pathobiont, can cause infections, including pneumonia, invasive pneumococcal disease, meningitis, and sepsis (4, 5). As novel antibiotic approval rates have declined and the frequency of antibiotic-resistant strains of *Spn* has surged, addressing this pathogen is imperative (6). Several endolysins that target *Spn*, such as Cpl-1 (7) and Pal (8), have shown efficacy both *in vitro* and *in vivo*. Many endolysins share a common cysteine, histidine-dependent amidohydrolase/peptidase (CHAP) domain (9). Several CHAP-containing endolysins with antimicrobial activity against *Staphylococcus aureus*, including N-Rephasin (SAL200) and Exebacase (CF-301), have been investigated in human clinical trials (3). However, no naturally occurring pneumococcal endolysin with a CHAP domain has been described. We have recently identified 76 putative pneumococcal endolysins from uncultured bacteriophage genomes by searching for a consensus sequence found in the cell-binding domain region of Cpl-1, Pal, and LytA (a *Spn* autolysin). One candidate endolysin, SP-CHAP, contains a CHAP domain and forms a dimer in the presence of choline, suggesting increased binding to the pneumococcal cell wall (10). Here, we characterize the biochemical and antimicrobial properties of SP-CHAP and benchmark it against Cpl-1.

### Biochemical characterization of SP-CHAP

**Fig 1:**
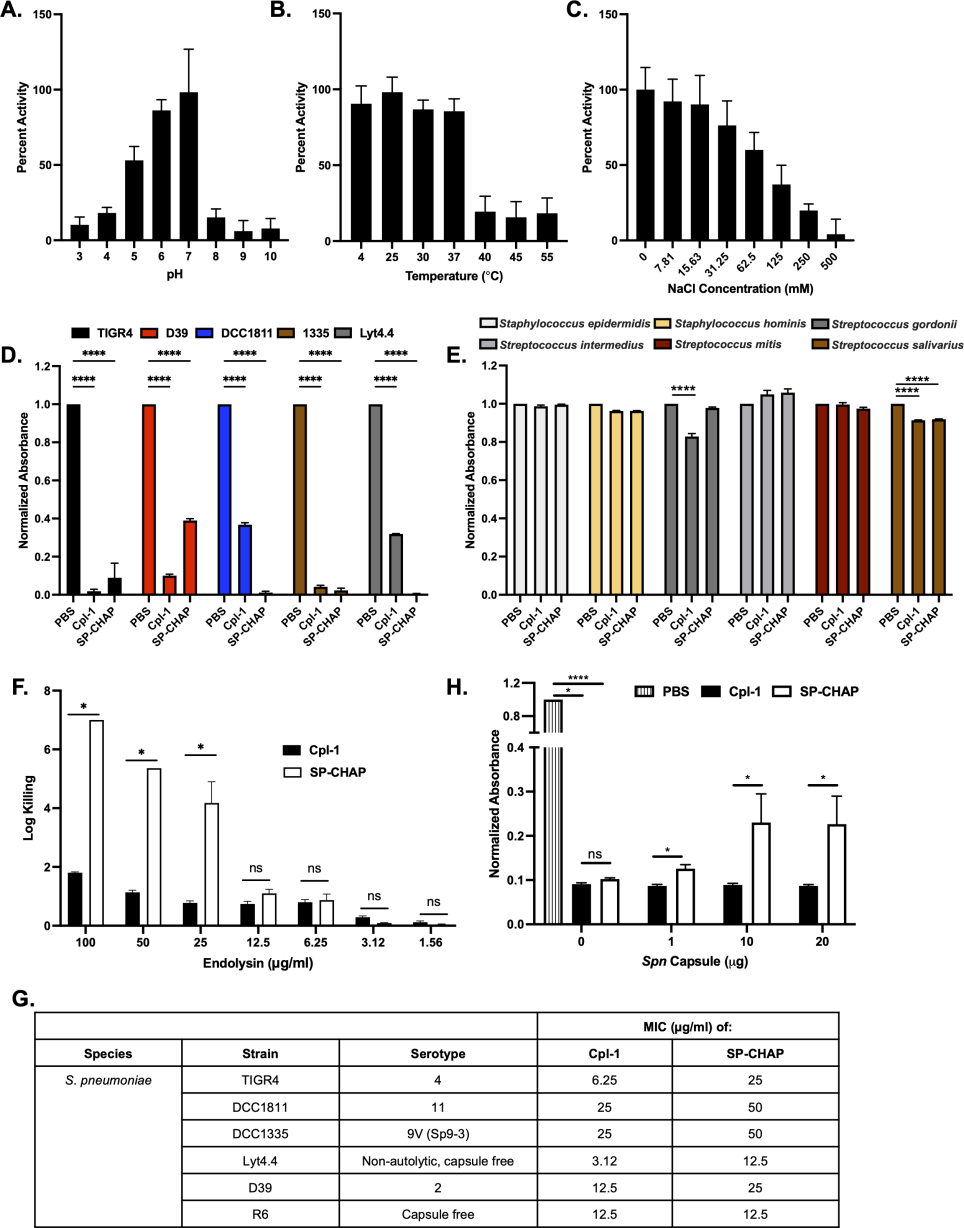
Endolysin SP-CHAP is highly active against planktonic-grown pneumococci, and its activity is modulated by capsule polysaccharide availability. Biochemical characterization of optimal conditions for SP-CHAP activity. The role of pH (**A**), temperature (**B**), and salt (**C**) on SP-CHAP lytic activity against *Spn* DCC1811. Lytic activity of 50 µg/mL Cpl-1 and SP-CHAP on (**D**) *Spn* strains TIGR4, D39, DCC1811, 1335, and Lyt4.4 or (**E**) the commensal staphylococcal bacteria (*Staphylococcus hominis* and *Staphylococcus epidermidis*) and commensal streptococcal bacteria (*Streptococcus gordonii*, *Streptococcus intermedius*, *Streptococcus mitis*, *and Streptococcus salivarius*) as measured by OD_600_. (**F**) Lytic activity of SP-CHAP and Cpl-1 at various concentrations on *Spn* Lyt4.4 as measured by the colony-forming unit assay. Data are reported as the log-fold killing compared to untreated controls. (**G**) Minimum inhibitory concentration (MIC) (μg/mL of endolysin) of SP-CHAP and Cpl-1 on *Spn* strains TIGR4, DCC1811, DCC1335, Lyt4.4, D39, and R6. (**H**) Lytic activity of 50 µg/mL Cpl-1 and SP-CHAP on *Spn* strain Lyt4.4 after incubation with purified pneumococcal capsular polysaccharide. Experiments were done in triplicate, and the error bars represent the standard deviations. For two-way ANOVA with Dunnett’s multiple comparisons test, asterisks denote the level of significance observed: ns, not significant; *P ≤ 0.05, **P ≤ 0.01, ***P ≤ 0.001, and ****P ≤ 0.0001.

To determine the optimum pH for SP-CHAP enzymatic activity, we measured lytic activity over a pH range of 3.0–10.0 in a turbidity reduction assay (Fig. 1A). The optimum SP-CHAP pH of 6.0–7.0 is similar to that seen for Cpl-1, Pal, and LytA (11). Next, the thermodynamic stability of SP-CHAP was tested by incubating the endolysin at temperatures ranging from 4°C to 55°C. SP-CHAP displayed lytic activity on *Spn* up to 37°C (Fig. 1B) with a dramatic decrease in activity at 40°C, 45°C, and 55°C, suggesting a melting temperature (*T*_*m*_) between 37°C and 40°C. This *T*_*m*_ is consistent with melting temperatures of other pneumococcal endolysins, such as 43.5°C for Cpl-1 (12), 37°C for Pal (13), and 43.9°C for Cpl-7 (14). The antibacterial effectiveness of several endolysins has been shown to increase with the addition of NaCl (15). However, using a constant concentration of 25 µg/mL SP-CHAP, we observed an inverse correlation between NaCl concentration and enzymatic activity (Fig. 1C). These results suggest the importance of ionic interactions for the bacterial surface, a phenomenon that has also been noted in the *Bacillus*-specific endolysins PlyP56, PlyN74, and PlyTB40 (16).

### SP-CHAP has greater lytic activity than Cpl-1 and activity can be partially inhibited by pneumococcal capsule

To benchmark SP-CHAP against Cpl-1, we evaluated their bacteriolytic activity against several pneumococcal serovars and oral or nasal commensal organisms. While both SP-CHAP and Cpl-1 killed all pneumococcal strains tested, SP-CHAP appeared to be more effective against three of the five strains (Fig. 1D). Of note, the two endolysins did not kill the commensal organisms tested (Fig. 1E). Next, we performed a colony-forming unit (CFU) assay to quantify the lytic potential of SP-CHAP. With a 1 h treatment, a dose-dependent effect of SP-CHAP lytic activity on *Spn* Lyt4.4 was observed at concentrations from 1.56 to 100 µg/mL (Fig. 1F). Treatment of *Spn* with 100 µg/mL SP-CHAP resulted in 7 log_10_ decrease in Lyt4.4 CFU, while Cpl-1 caused only a 2-log reduction at the same concentration. However, there was not a significant difference in activity between SP-CHAP and Cpl-1 at 12.5–1.56 µg/mL. To further quantify SP-CHAP activity, we performed standard minimum inhibitory concentration (MIC) assays. Cpl-1 displayed higher antimicrobial activity with a lower MIC than SP-CHAP against several *Spn* strains tested (Fig. 1G). We attribute these observed disparities in the activity of Cpl-1 and SP-CHAP to differences in the assay methodologies, as previously documented for other cell wall hydrolases (17). Specifically, the turbidity reduction assay and the log-fold killing assays involved incubating the enzymes with bacteria for 30 min or 1 h, primarily assessing bacteriolytic activity. In contrast, the MIC assays require overnight incubation of enzymes with bacteria, primarily assessing bacteriostatic activity.

These results highlighted variations in SP-CHAP activity against the encapsulated D39 strain and its capsule-free derivatives, R6 and Lyt4.4. Consequently, we hypothesized that the presence of capsular polysaccharide may modulate SP-CHAP activity. This hypothesis stems from previous findings demonstrating that capsule shedding is a known pneumococcal response to antimicrobial peptides (18) and that the presence of a capsule in *Klebsiella pneumoniae* is linked to reduced endolysin activity (19). Pre-incubation of the purified capsular polysaccharide with the endolysins 30 min prior to and throughout the duration of a lytic assay resulted in a partial yet significant reduction in the lytic activity of SP-CHAP, but this effect was not observed with Cpl-1 (Fig. 1H).

### SP-CHAP has greater activity than Cpl-1 against biofilm pneumococci

**Fig 2:**
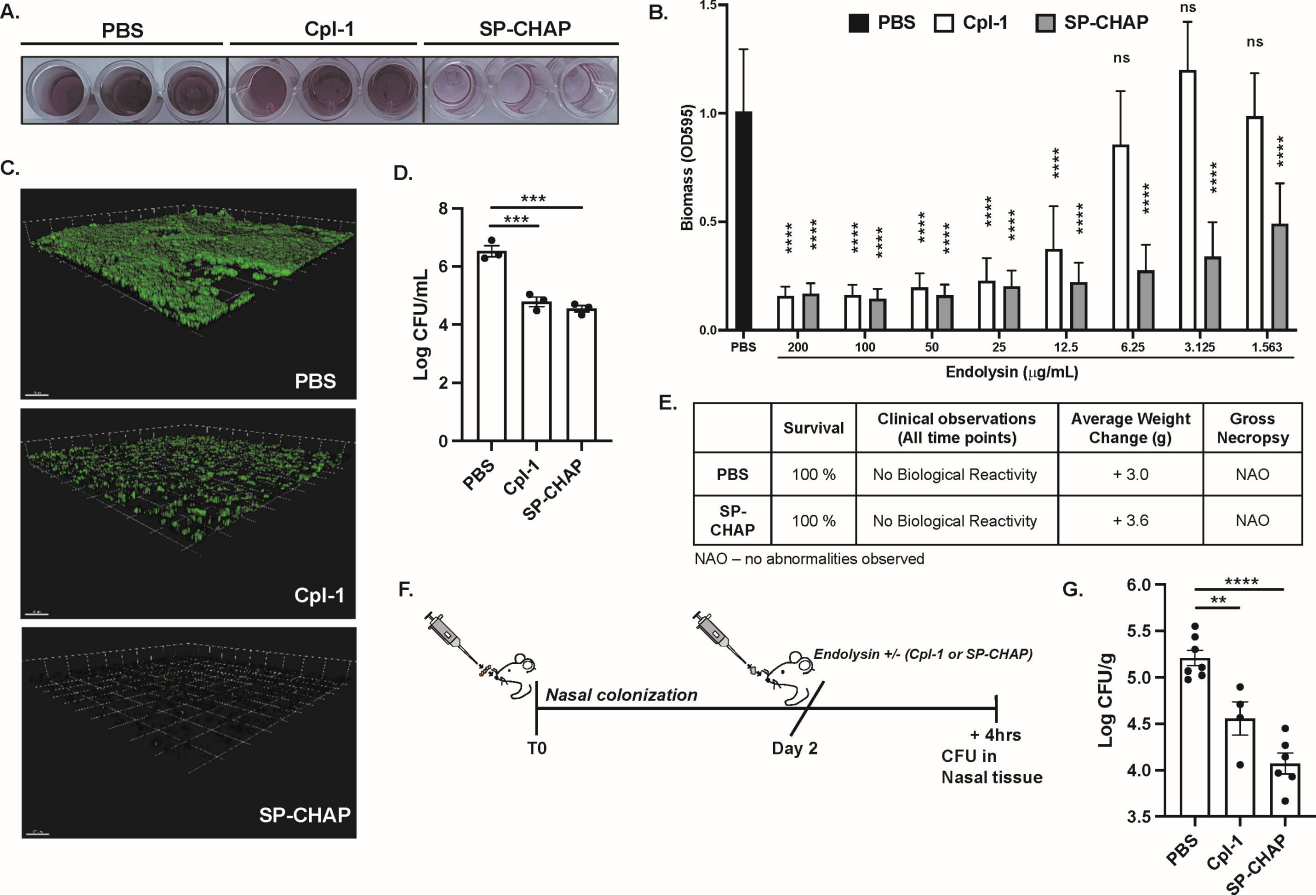
SP-CHAP reduces pneumococcal biofilm biomass *in vitro* and nasopharyngeal colonization *in vivo*. (**A**) Disruption of *Spn* Lyt4.4 biofilms by PBS,or 1.56 µg/mL Cpl-1 or SP-CHAP, as visualized by crystal violet staining. (**B**) Quantification of biomass measured by OD_595_ of crystal violet-stained biofilms. SP-CHAP displayed biofilm eradication ability at all concentrations tested, while Cpl-1 removed biofilms at concentrations as low as 12.5 µg/mL. Statistical analysis denotes differences between PBS and endolysin-treated wells. (**C**) 630× confocal microscopy consisting of nineteen 0.5 µm slices of *Spn* biofilms treated with PBS, Cpl-1, or SP-CHAP. Scale bar = 10 µm. (**D**) Viability of *Spn* Lyt4.4 biofilms after treatment with 20 µg/mL Cpl-1 or SP-CHAP for 1 h as measured by the CFU assay. Data are reported as the Log CFU/mL of recovered bacteria. (**E**) Acute systemic toxicity in mice intraperitoneally challenged with vehicle or SP-CHAP. (**F**) Sketch of mouse colonization model and (**G**) Log CFU per gram of nasopharyngeal tissue from mice infected with *Spn* strain TIGR4 and treated with 60 µg of endolysin 48 h post-colonization and samples collected 4 h post-treatment. Samples were tested with two-way ANOVA or one-way ANOVA Kruskal-Wallis test with Dunn’s multiple-comparisons post-test. Asterisks denote the level of significance observed: ns, not significant; *P ≤ 0.05, **P ≤ 0.01, ***P ≤ 0.001, and ****P ≤ 0.0001.

*Spn* can form biofilms to promote colonization of the nasopharynx and to improve antibiotic resistance (20). Using a static biofilm model, we observed that SP-CHAP was highly efficient at eradicating pneumococcal biofilms when compared to Cpl-1 (Fig. 2A). Furthermore, when 24-h mature biofilms were treated with SP-CHAP for 1 h, it caused a dose-dependent decrease in biofilm volume at all concentrations tested, but Cpl-1 only dispersed biofilms at 12.5 µg/mL and higher concentrations (Fig. 2B). Supporting these observations, confocal imaging of biofilms demonstrated that biofilms treated with endolysins exhibited thinner structures compared to the control group, with SP-CHAP causing a greater reduction in biofilm thickness than Cpl-1 (Fig. 2C). These anti-biofilm properties were mirrored by similar decreases in *Spn* viability within the biofilms (Fig. 2D).

### SP-CHAP is not toxic to the host but efficiently decolonizes the nasopharynx *in vivo*

Using a model of toxicity, we evaluated the systemic response to SP-CHAP following intraperitoneal injection into mice. Using a total injection of 600 µg SP-CHAP or 10× the dose used in the decolonization studies below, we observed that none of the test animals exhibited any clinical signs of distress throughout the 3-day observation period. All animals from the test group gained weight, and no abnormalities were noted during gross necropsy (Fig. 2E). Finally, to assess the effectiveness of SP-CHAP as a decolonizing agent *in vivo*, we used a mouse *Spn* nasopharyngeal colonization model (Fig. 2F). We observed that intranasal administration of 60 µg SP-CHAP per mouse significantly reduced *Spn* CFUs in the nasal tissue, and this was slightly more pronounced than the effect of Cpl-1 (Fig. 2G). Of note, previous studies have demonstrated that intranasal delivery of 100 µg of penicillin was inadequate to decrease nasopharyngeal colonization by *Spn* (21), highlighting the advantages of endolysins over conventional antibiotics.

### Conclusion

In summary, our results indicate that SP-CHAP is a non-host toxic, highly efficient endolysin with the ability to lyse *Spn* and decolonize biofilm-grown bacteria both *in vitro* and *in vivo*. Evidence that capsule can reduce the activity of SP-CHAP during planktonic growth also establishes a novel observation as a possible mechanism for pneumococci to protect themselves against this class of antimicrobial. Taken together, our data establish the initial therapeutic capabilities of SP-CHAP and identify a potential mechanism employed by planktonic *Spn* to counter endolysin activity.

## MATERIALS AND METHODS

### Bacterial strains and growth conditions

*Streptococcus pneumoniae* was grown in static culture at 37°C with 5% CO_2_ in Todd Hewitt broth supplemented with 1% yeast extract (THY) or on THY agar plates. The *Spn* strain R6 is a capsule-free variant derived from strain D39, a serotype 2 strain (Fig. 1G). Strain Lyt4.4 represents a transformed version of the R6 strain where the major autolysin, LytA, has been inactivated, as previously described (22). Thus, Lyt4.4 is both capsule-free and LytA deficient. Due to the autolytic activity that *Spn* normally display in overnight cultures, in biofilms, and when exposed to capsule shedding, *Spn* Lyt4.4 was used for these experiments. Commensal organisms, *Staphylococcus hominis* SK119, *Staphylococcus epidermidis* SK135, *Streptococcus mitis* F0392, *Streptococcus gordonii* PK2565, *Streptococcus intermedius* PK2821, and *Streptococcus salivarius* ATCC 27945 were grown in Brain Heart Infusion media (Difco) at 37°C. *Escherichia coli* DH5α and BL21 (DE3) strains were grown in Luria-Bertani (LB) broth or agar. All strains were stored at −80°C.

### Cloning

Cpl-1 (GenBank: NP_044837.1) and SP-CHAP (OR644363) used in this study were codon-optimized for *E. coli* expression and synthesized (GeneArt). A 6×-His tag was added to the C-terminal end. The constructs were sub-cloned into the pBAD24 expression vector, and the sequence was confirmed (Psomagen, Rockville, MD, USA) before being transformed into *E. coli* BL21 (DE3) for protein expression. The ApE program (University of Utah) was used for DNA sequence analysis and manipulations.

### Protein expression and purification

Overnight cultures of *E. coli* BL21 (DE3) containing the plasmid of interest were sub-cultured 1:100 into 1.5 L baffled flasks containing LB supplemented with 100 µg/mL antibiotic and grown at 37°C. Expression was induced while cells were in the mid-log phase with 0.25% arabinose and incubated overnight at 18°C. Cells were harvested (5,000 × *g*, 15 min, 4°C), resuspended in 20 mL of lysis buffer (PBS and 10 mM imidazole, pH 7.4), and disrupted by freeze-thawing (−80°C–4°C). This was followed by sonication on ice (power level 6 and duty cycle 30%). Insoluble cell debris was removed by centrifugation (12,000 × *g*, 1 h, 4°C), and the cell lysate containing the 6×-His tagged protein was loaded onto Ni-NTA resin (HisPur Ni-NTA Resin, Thermo Scientific) in a gravity flow column and eluted in 4 mL fractions using an imidazole step gradient in PBS buffer (20, 50, 100, 250, and 500 mM imidazole). Protein purity was analyzed on an SDS-PAGE gel. Samples were additionally purified using a Sephacryl S-200 column and an ÄKTA Pure 25 L system (both from Cytiva). Samples were pooled and concentrated using a protein concentrator with a 10 kDa molecular weight cutoff (Amicon Ultra 15 mL centrifugal filters, Millipore Sigma).

### Turbidity reduction assay

Bacteriolytic activity was measured by means of a turbidity reduction assay as previously described (23). Overnight cultures of *Spn* were centrifuged, and the pellets were washed twice and resuspended in PBS to a final optical density at 600 nm (OD_600_) of 0.9–1.2. Cells were mixed with SP-CHAP or Cpl-1 with a final protein concentration of 100 µg/mL. The OD_600_ was monitored by the use of a microplate spectrophotometer (SpectraMax ABS; Molecular Devices, USA) every 15 s for 30 min at 37°C. Lytic activity was quantified as the reduction in turbidity and measured as the difference in OD_600_ between PBS control-treated cells and cells that were treated with SP-CHAP or Cpl-1 over 30 min.

### Colony-forming unit assay

CFU counting was also conducted to quantify antimicrobial activity. Sterile protein at a final concentration of 100 µg/mL was used for this assay. Briefly, 100 µL of protein was mixed with 100 µL of *Spn* Lyt4.4 in PBS in a 96-well plate and incubated at 37°C for an hour. Then, five 10-fold dilutions in PBS were made, and 10 µL from each dilution was plated on THY agar plates, air-dried, and placed in a 37°C incubator overnight. The CFUs were counted, and the data were reported as the log-fold killing compared to the untreated control. Each turbidity reduction assay and CFU counting assay were performed in triplicate on three separate days.

### Minimum inhibitory concentration assay

One hundred microliters of an overnight *Spn* culture was diluted in 10 mL of THY media (1:100) with 50 µM catalase. Ten twofold dilutions of endolysins were prepared, starting at 100 µg/mL. Following this, 100 µL of the diluted culture was mixed with 100 µL of each endolysin treatment at every concentration in triplicate, using a 96-well plate. The plate was then incubated overnight in a 37°C non-shaking incubator and observed the following day to identify the lowest concentration at which there was a clear well with no bacterial growth. The OD_600_ was measured for accuracy using a SpectraMax ABS plate reader.

### Endolysin inhibition assay

Overnight cultures of *Spn* Lyt4.4 were centrifuged, and the pellets were washed twice and resuspended in PBS. SP-CHAP or Cpl-1 with a final protein concentration of 50 µg/mL were mixed with 1, 10, or 20 µg of purified *Spn* serotype 4 capsular polysaccharide (76855, SSI Diagnostica) and incubated at room temperature for 15 min. *Spn* cultures were then challenged with the endolysin-capsule mixtures, and after a 30-min incubation time, lytic activity was quantified via the turbidity reduction assay described above.

### Biofilm assay

*Spn* Lyt4.4 were grown in THY media to an OD_600_ of 0.5–0.6, sedimented by centrifugation, and resuspended in an equal volume of fresh THY media. The cells were then diluted 1:2 into a fresh 15 mL tube, and 200 µL of this was dispensed into each well of a Costar 96-well flat-bottom polystyrene plate (Corning). The plate was then incubated at 37°C overnight. The next day, the media were carefully pipetted out from each well, and the biofilm formed was gently washed twice with PBS to remove unattached cells. Twofold serial dilutions of SP-CHAP or Cpl-1 were added to treatment wells, and the plate was incubated at 37°C for 1 h. The liquid was then pipetted out, and the wells were washed with distilled water and left to air dry. Biofilms were stained with 50 µL of a 0.2% crystal violet solution for 15 min and subsequently washed three times with distilled water and air-dried. The biofilm was solubilized in 10% SDS to extract crystal violet from the biomass, transferred to a fresh 96-well plate, and absorbance quantified at OD_595_ using a SpectraMax M5 plate reader. Alternately, identically treated biofilms with 20 µg/mL Cpl-1 or SP-CHAP were scrapped from wells, pipetted to break up the biofilms, serially diluted, and incubated overnight as described above in the colony-forming unit assay to enumerate *Spn* viability.

### Confocal microscopy

*Spn* biofilms were grown in chamber slides (Nunc Lab-Tek chamber slides, Thermo Fisher Scientific) following previously established procedures (24). One hundred microliters of a 100 µg/mL solution of each endolysin was added and allowed to incubate at room temperature for 1 h. After washing with PBS, the biofilms were stained using the Live/Dead BacLight stain (Thermo Fisher Scientific) before being examined by confocal microscopy. Mounted samples were visualized using a Zeiss LSM800 confocal microscope at 63× with 1× digital zoom. Z-stacks of each biofilm sample were acquired using the 488 nm laser and 500–619 nm detection filter with a constant 0.5 µm *Z*-axis interval for 19 slices. 3D images of the biofilms were rendered using the normal shading mode of Imaris 9.0.1 software (Oxford Instruments). All acquisition and image settings were set the same across all samples.

### Acute systemic toxicity testing

Acute systemic toxicity testing was done by Pacific BioLabs. Briefly, SP-CHAP was prepared according to ISO 10993-12 and Pacific BioLabs internal SOPs (Pacific BioLabs, Hercules, CA, USA). Saline without SP-CHAP was used as the vehicle (negative) control. Ten Swiss-Webster albino mice were randomly assigned to SP-CHAP or vehicle groups. Each animal was injected intraperitoneally with 200 µL of saline or SP-CHAP at a concentration of 3 mg/mL. The animals were observed for biological reactivity immediately after and at 4 h ± 15 min, 24 ± 2 h, 48 ± 2 h, and 72 ± 2 h following injection. Animals were weighed at 24 ± 2 h, 48 ± 2 h, and 72 ± 2 h following injection. At the end of the study, animals were euthanized, and gross necropsy was performed.

### Mouse colonization studies

The mouse colonization experiments were similar to those previously described (20, 25). Briefly, mice were infected intranasally with 1 × 10^5^ CFU *Spn* TIGR4. After 48 h, 25 µL of endotoxin-free, filter-sterilized PBS (vehicle), 1.2 mg/mL Cpl-1, or 1.2 mg/mL SP-CHAP were delivered to each nostril. After an additional 4 h, mice were euthanized and nasopharyngeal tissue was excised, weighed, and homogenized. Homogenates were serially diluted and plated on blood agar plates to determine CFU per gram of tissue.

### Statistical analysis

Unless otherwise noted, all *in vitro* experiments had a minimum of three biological replicates, with ≥3 technical replicates. GraphPad Prism 8 (La Jolla, CA, USA) was used for statistical analyses. Comparisons between two cohorts at a single time point were calculated by the Mann-Whitney *U* test. Comparisons between groups of >2 cohorts or groups given multiple treatments were calculated by ANOVA with Tukey’s (one-way) or Dunnett’s (two-way) post-test or by Kruskal-Wallis *H* test with Dunn’s multiple comparison post-test, as determined by the normality of data groups. Repeated measures are accounted for whenever applicable.
